# Native habitat affinities predict fish invasions with post‐invasion habitat shifts

**DOI:** 10.1111/1365-2656.70293

**Published:** 2026-07-01

**Authors:** Shahar Chaikin, Tal Gavriel, Avery Deveto, Shahar Malamud, Jonathan Belmaker

**Affiliations:** ^1^ Department of Biogeography and Global Change National Museum of Natural Sciences CSIC Madrid Spain; ^2^ School of Zoology, Faculty of Life Sciences Tel Aviv University Tel‐Aviv Israel; ^3^ The Steinhardt Museum of Natural History Tel Aviv University Tel‐Aviv Israel

**Keywords:** biological invasions, marine fish, niche breadth, niche shift, species traits, stereo‐BRUVs

## Abstract

1. Identifying habitat affinities and demographic characteristics associated with high invasion potential is a key ecological challenge. However, testing invasion hypotheses exclusively in the invaded range is inherently flawed, as it conflates the intrinsic pre‐adaptations that allowed the species to invade with the habitat shifts they adopted post‐invasion.
2. To elucidate how invasion potential is related to habitat affinities and post‐invasion ecological dynamics, we investigated the unique paired‐basin system of the Red Sea and Eastern Mediterranean. We captured in situ realized niche data (e.g. abundance, depth limits and habitat affinities) using standardized stereo underwater video surveys across 179 populations. We then applied a three‐comparison framework to evaluate native‐range invasion potential, post‐invasion habitat shifts and recipient‐community integration.
3. Within the native Red Sea, future introduced species were distinct from non‐introduced natives primarily due to their lack of reliance on coral habitats, shallower minimum depth limits, and broader habitat and depth ranges.
4. Comparing the native and invaded ranges of introduced species revealed post‐invasion niche shifts. Rather than shifting uniformly, introduced species exhibited high habitat lability, successfully abandoning affinities for tropical substrates to utilize available Mediterranean habitats. Within the Mediterranean, introduced species were assimilated into the recipient community, becoming indistinguishable from natives in habitat use rather than occupying distinct habitats.
5. By synthesizing linear discriminant and mixed‐modelling approaches into a cross‐validated ensemble index, we prioritized specific Red Sea species as high‐risk candidates for future Mediterranean invasion. Ultimately, this in situ framework highlights that while specific native‐range habitat filters predict invasion potential, post‐invasion habitat lability means that relying solely on native affinities could severely underestimate their spread and impact in novel environments.

## Graphical abstract

Initial invasion success is gated by native pre‐adaptations, but post‐invasion establishment is characterized by habitat shifts. We demonstrate that forecasting risk based solely on native niches severely underestimates future ecological impacts, and we provide an empirically validated watchlist of future invaders.

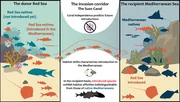

## תקציר

1. זיהוי זיקות לבית גידול ומאפיינים דמוגרפיים הקשורים לפוטנציאל פלישה גבוה מהווה אתגר אקולוגי מרכזי. עם זאת, בחינת השערות פלישה אך ורק באזור שאליו פלשו המינים היא פגומה מיסודה, שכן היא כורכת את ההתאמות המוקדמת הטבעיות שאפשרו למינים לפלוש, יחד עם השינויים בבית הגידול שאותם אימצו לאחר הפלישה.
2. כדי להבהיר כיצד פוטנציאל פלישה קשור לזיקות לבית גידול ולדינמיקה אקולוגית לאחר הפלישה, חקרנו את מערכת האגנים הצמודים הייחודית של ים סוף ומזרח הים התיכון. אספנו נתוני נישת בית גידול ממומשת בשטח באמצעות סקרי וידאו תת‐ימי סטריאוסקופי בקרב 179 אוכלוסיות. לאחר מכן, יישמנו מסגרת אבחנתית בעלת שלוש השוואות כדי להעריך את פוטנציאל הפלישה בסביבת המקור, את השינויים בבית הגידול לאחר הפלישה ואת ההשתלבות בחברת הדגים הקולטת.
3. בתוך סביבת המקור בים סוף, מינים (אשר לימים פלשו) נבדלו ממינים מקומיים שלא פלשו, בעיקר בשל חוסר התלות שלהם בבתי גידול אלמוגיים, גבולות עומק מינימלי רדודים יותר, וטווחי בית גידול ועומק רחבים יותר.
4. השוואה בין אזורי התפוצה המקוריים ואלו שאליהם פלשו המינים חשפה שינויי נישת בית גידול לאחר הפלישה. במקום להשתנות באופן אחיד, המינים הפולשים הציגו גמישות גבוהה בבחירת בית גידול, תוך נטישה מוצלחת של הזיקה למצעים טרופיים לטובת ניצול בתי גידול זמינים בים התיכון. בתוך הים התיכון, המינים הפולשים נטמעו בחברת הדגים הקולטת והפכו לבלתי ניתנים להבחנה מהמינים המקומיים באשר לשימוש בבית הגידול, במקום לאכלס בתי גידול נפרדים.
5. על ידי שילוב שלlinear discriminant analysis וmixed‐modelling לכדי מדד משולב בעל אימות צולב (cross‐validated ensemble index), תעדפנו מינים ספציפיים מים סוף כמועמדים בסיכון גבוה לפלישה עתידית לים התיכון. בסופו של דבר, עבודת שטח זו מדגישה כי בעוד שמסנני בית גידול ספציפיים בסביבת המקור מנבאים את פוטנציאל הפלישה, הגמישות בבחירת בית הגידול לאחר הפלישה משמעותה שהסתמכות בלעדית על זיקות המקור עלולה להוביל להערכת חסר חמורה של התפשטותם והשפעתם של המינים בסביבות חדשות.

## الملخص

يعد تحديد الألفة البيئية والخصائص الديموغرافية المرتبطة بالقدرة العالية على الاجتياح البيئي أحد التحديات الرئيسية في علم الأحياء ومع. البيئي ذلك، فإن اختبار فرضيات الاجتياح حصرًا داخل النطاق المتاح ينطوي على خلل جوهري، كونه يخلط بين التكيفات الذاتية المسبقة التي مكنت النوع من الانتشار، وبين التحولات البيئية التي تبناها بعد عملية الاجتياح. وبهدف توضيح كيفية ارتباط الإمكانيات الاجتياحية بالألفة البيئية والديناميكيات الإيكولوجية لما بعد الاجتياح، قمنا بدراسة النظام الحوضي المزدوج الفريد للبحر الأحمر وشرق البحر الأبيض حيث. المتوسط رصدنا بيانات المناصب البيئية الفعلية في مواقعها الوفرة: مثل (الطبيعية العددية، حدود العمق، والألفة باستخدام) البيئية التوثيق المرئي تحت الماء والذي يشمل ١٧٩ فئة بعد. أحيائية ذلك، طبقنا إطارًا تحليليًّا ثلاثي المقارنة لتقييم كل القدرة: من الاجتياحية داخل النطاق الأصلي، والتحولات البيئية لما بعد الاجتياح، ومدى الاندماج في الفئة المستقبِلة. وقد أظهرت النتائج داخل النطاق الأصلي للبحر الأحمر أن الأنواع المرشحة للإدخال مستقبلًا كانت تتميز عن الأنواع الأصلية غير المجتاحة بشكل رئيسي بعدم اعتمادها على بيئات الشعاب المرجانية، وبوجود حدود عميقة أكثر ضحالة، فضلًا عن اتساع نطاقاتها البيئية والعميقة. وعند مقارنة النطاقات الأصلية بالنطاقات المجتاحة للأنواع المُدخَلَة، انكشفت لنا تحولات في المناصب البيئية لما بعد فبدلًا. الاجتياح من أن تتحول هذه الأنواع بشكل نمطي موحد، أبدت الأنواع المُدخَلَة مرونة بيئية عالية، إذ نجحت في التخلي عن ألفتها للبيئات الاستوائية لتستغل البيئات المتاحة في البحر أما. المتوسط داخل البحر المتوسط، فقد تمت مماثلة الأنواع المُدخَلَة واستيعابها ضمن الفئة المستقبِلة، حيث أصبحت غير قابلة للتمييز عن الأنواع الأصلية من حيث استخدام البيئة، بدلًا من شغل بيئات منفصلة. ومن خلال الجمع المنهجي بين مناهج التحليل التمييزي الخطي وتحليل التأثيرات المختلطة ضمن مؤشر تجميعي خاضع للتحقق المتبادل، قمنا بتصنيف وترتيب أولوية أنواع معينة من البحر الأحمر بوصفها كائنات عالية الخطورة لاجتياح البحر المتوسط مستقبلًا.

## 1 INTRODUCTION

**FIGURE 1:**
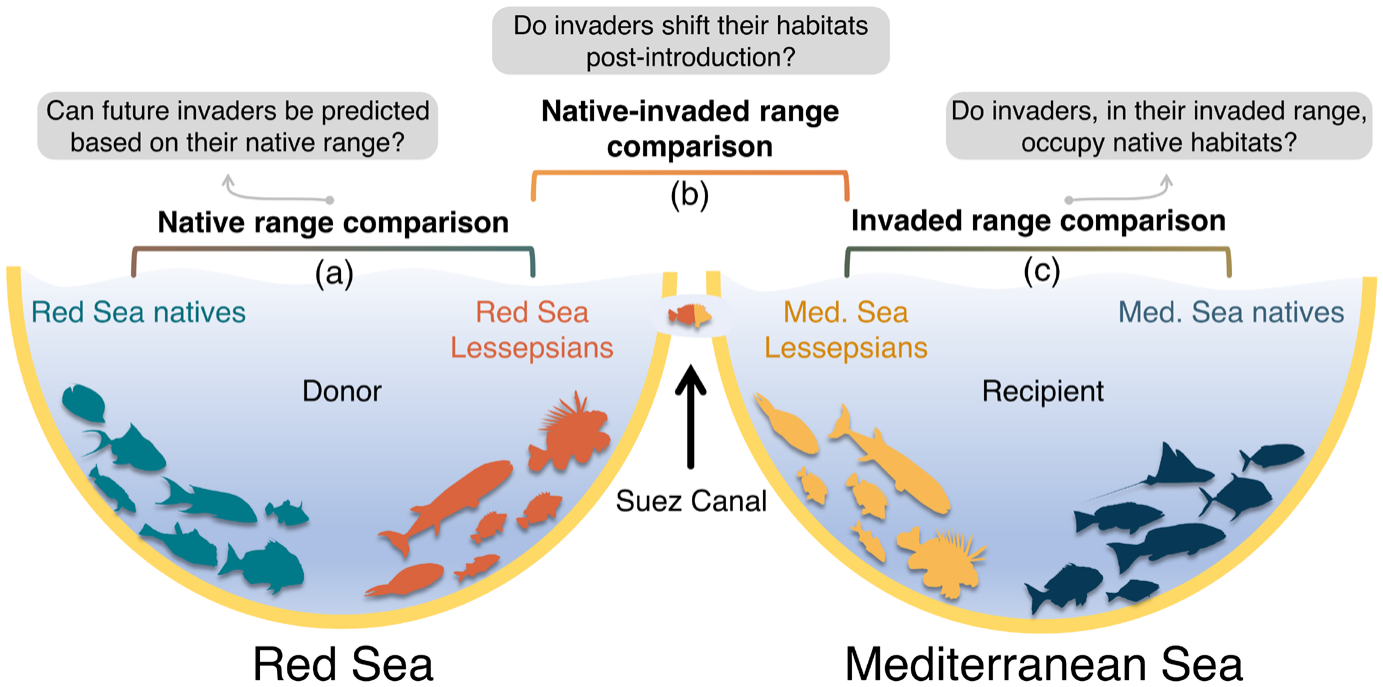
Conceptual framework of the multi‐comparison approach used to characterize Lessepsian invasion. (a) Native‐range comparison: Tests for source‐pool filtering by contrasting the characteristics of non‐introduced Red Sea species (light blue) against the introduced species pool (Red Sea populations of Lessepsian species; red). (b) Native‐invaded‐range comparison: Quantifies post‐introduction habitat and abundance shifts by comparing source populations (Red Sea Lessepsians) with introduced populations in the Mediterranean (Mediterranean Sea Lessepsians; yellow). (c) Invaded‐range comparison: Evaluates community integration by assessing the habitat and abundance patterns of introduced species relative to the native Mediterranean assemblage (dark blue). Grey boxes highlight key questions.

The Anthropocene is characterized by the unprecedented redistribution of species, encompassing human‐mediated species introductions and climate‐driven range expansions—both of which constitute contemporary biological invasions (Carlton & Schwindt, 2026; see Table S1 for standardized glossary). While redistributions are often attributed to ocean warming (Diez et al., 2012; King et al., 2021; Lenoir et al., 2020; Sorte et al., 2013), temperature alone rarely dictates invasion (Shea & Chesson, 2002). The ability of a species to form a self‐sustaining population outside its historical range also depends heavily on non‐climatic factors, such as the availability of suitable habitat and overall ecological generalism (Higgins & Richardson, 2014; Stachowicz et al., 2002). Specifically, when efforts are made to identify habitat affinities and ecological characteristics driving invasion potential, studies often evaluate introduced species within their newly invaded range. Crucially, testing invasion hypotheses exclusively in the invaded‐range risks conflating post‐invasion habitat shifts with intrinsic pre‐adaptations that actually facilitated the initial establishment (Gallien & Carboni, 2017). Overcoming this limitation requires a holistic approach encompassing (Figure 1): (1) *native‐range comparisons* to identify intrinsic invasion potential, (2) *native‐invaded‐range comparisons* to quantify habitat shifts and (3) *invaded‐range comparisons* to assess the level of integration with the recipient community.

The first pillar of this framework, the native‐range comparisons, tests for ecological filtering at the source. By comparing species that have become introduced elsewhere against non‐introduced species within the same native pool, we can isolate the intrinsic habitat affinities associated with a high probability of invasion (van Kleunen et al., 2010). For example, the ‘niche breadth hypothesis’ posits that generalists are inherently more capable of persisting in novel environments (Higgins & Richardson, 2014; Hutchinson, 1957; Levins, 1968). However, empirical studies focusing on this hypothesis are heavily biased towards plant studies (Jeschke et al., 2012; Lowry et al., 2012; Pysek et al., 2008), which may not accurately capture the invasion dynamics of highly mobile animals like marine fishes. In addition to niche breadth, high abundance in the native range may serve as a proxy for high propagule pressure and greater dispersal capabilities, increasing the likelihood of successful establishment (Table 1; Lockwood et al., 2005; Simberloff, 2009). Identifying these non‐climatic, source‐pool predictors is vital; as although climate change may drive poleward movements, the ability of species to establish in novel environments may depend on their habitat breadth.

**TABLE 1:** Predicted ecological differences for the native‐range comparison.

Component	Predictions for Red Sea Lessepsians	Explanation	Refs.	Variables measured in this study
Depth	Shallower minimum depth, larger depth range	The Suez Canal is shallow; thus, species capable of persisting in shallow habitats may pass the canal with higher probability. Species with larger depth ranges may have improved settlement opportunities	Arndt and Schembri (2015)	Depth range/ Min. depth
Habitat breadth	Wider habitat breadth	Species with large habitat breadth may have higher probabilities of establishing populations in new environments	Warren et al. (2001) and Sunday et al. (2015)	Standardized Hurlbert's niche breadth
Habitat affinity	Affinity towards unconsolidated habitats	Continuous cover of sandy habitats from the Gulf of Suez to the Levantine Basin. Lack of developed coral reefs in the Mediterranean	Golani and Ben‐Tuvia (1989) and Golani (1993)	Frequency of occurrence on habitat types
Abundance and commonness	Higher relative abundance and occupancy	Higher abundance may indicate higher propagule pressure and thus increase colonization success	Crawley (1987), Williamson and Fitter (1996b) and Lockwood et al. (2005)	MaxN/commonness
Temperature	Broader thermal range	Species occurring in wider local thermal extremes are more likely to survive transport through the Suez Canal and establish in the temperate Mediterranean	Bates et al. (2013), Belmaker et al. (2013) and Kelley (2014)	Temperature range

*Note*: Hypotheses outline the habitat affinities, abundance and temperature metrics expected to distinguish successful introduced species (Lessepsian species) from non‐introduced species within the Red Sea source pool.

While native‐range comparisons predict who might invade, they cannot reveal how species adapt to novel conditions post‐invasion. Native‐invaded‐range comparisons address this gap by quantifying ecological shifts between a species' historical source population and its newly established population (Bates & Bertelsmeier, 2021; Guisan et al., 2014; Parravicini et al., 2015). For warm‐adapted species shifting into temperate regions, adapting to novel thermal regimes, altered habitats and different resource availability may be critical for establishment. Conversely, lack of niche shift may support the pre‐adaptation of introduced species to their novel range. This conservatism may manifest as either a full retention of the native niche or by occupying a subset of their historical niche conditions in the new range (Liu et al., 2025). Evidence for post‐invasion niche dynamics remains mixed, with some studies arguing for niche conservatism (Beukema et al., 2018; Liu et al., 2020; Petitpierre et al., 2012; Strubbe et al., 2013, 2015). Quantifying these shifts is essential because if introduced species retain their native niche in the invaded range, our knowledge from the native range can be reliably used to predict their potential spread and impact in the new environment.

Finally, invaded‐range comparisons evaluate how introduced species integrate into the recipient ecosystem. This comparison is essential to estimate, as if introduced species are distinct in habitat use from the native community, it would facilitate coexistence through the separation of niches, while similar habitat use may suggest a high potential for competition (Darwin, 1859; MacArthur, 1970). By comparing the habitat associations of introduced species against the native community in the invaded range, we can test whether introduced species insert themselves into vacant habitats or overlap with historical natives (Givan et al., 2017; Ordonez et al., 2010; Steger et al., 2021). This comparison determines whether the invasion broadens the habitat diversity of the recipient community or leads to elevated potential for competitive exclusion.

We use an ‘historical experiment’ to deploy this three‐comparison framework (Figure 1). The opening of the Suez Canal in 1869 physically connected the subtropical Red Sea to the temperate Mediterranean Sea, facilitating the expansion of Lessepsian migrants—defined as a geographically specific subset of introduced species, strictly referring to the influx of Red Sea species into the Mediterranean (Por, 1978)—including over 120 fish species (Azzurro et al., 2022; Galanidi et al., 2023; Golani, 2021; Kovacic et al., 2021; Rilov & Galil, 2009). This system provides a model of both human‐mediated species introductions and climate‐driven range expansions (Ben Rais Lasram et al., 2010; Bianchi & Morri, 2003; Coll et al., 2010). By breaching a geographical barrier, the Suez Canal opened a previously closed tropical dead‐end, allowing species to spill into the temperate Mediterranean, where ocean warming may increasingly accommodate their continued expansion (Schickele et al., 2021). The well‐defined species pools and extensive historical records make this paired‐basin system ideal for testing hypotheses related to biological invasions. While previous studies have identified shifts in diet (Tsadok et al., 2023) or body size (Pickholtz et al., 2018; Ulman et al., 2022) for select Lessepsian species, or highlighted broad trait differences using species‐level data (Arndt et al., 2018; Belmaker et al., 2013; Givan et al., 2017), a comprehensive evaluation across all three comparisons using in situ collected habitat affinity estimates is lacking.

In this study, we surveyed fish assemblages across the Northern Red Sea and Eastern Mediterranean Sea using standardized Baited Remote Underwater stereo‐Video systems (stereo‐BRUVs). We collected ecological data (e.g. abundance, depth limits, habitat breadth and habitat affinities) for basin‐specific populations of each species. In total, this comprised 126 Red Sea and 53 Mediterranean Sea populations, including nine species where populations were recorded in both basins. By capturing in situ data, we systematically apply our multi‐comparison framework to: (1) test whether specific habitat affinities distinguish known introduced species from non‐introduced species in the native pool (i.e. source filtering); (2) quantify post‐invasion habitat shifts; and (3) evaluate the ecological distinctiveness of established Lessepsian species relative to the native Mediterranean community. Ultimately, by comparing donor and recipient environments, this framework provides novel insights into the dynamics of biological invasions, improving our ability to anticipate future range expansions.

## 2 MATERIALS AND METHODS

### 2.1 Study sites

**FIGURE 2:**
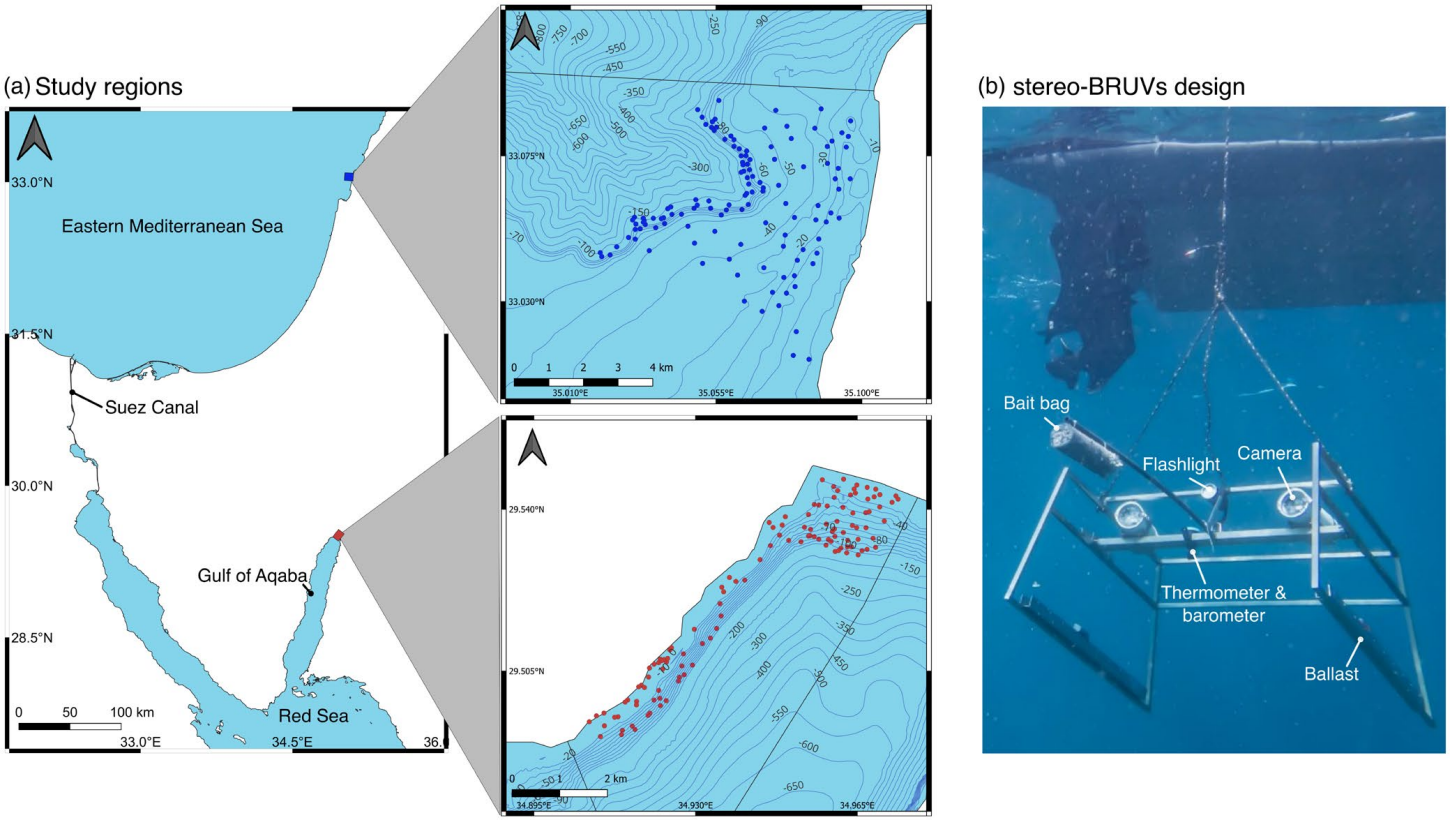
Study regions and the stereo‐BRUVs design. (a) Map illustrating the two sampling regions: The Northern Red Sea (red indicates sample locations; *n* = 123 deployments) and the Eastern Mediterranean Sea (blue indicates sample locations; *n* = 124). (b) Overview of the stereo‐BRUVs unit during deployment. Each system comprises two pre‐calibrated video cameras mounted on a base bar, a bait bag attached to a rigid arm, artificial lighting, temperature and pressure sensors, and ballast. Figure modified from Chaikin and Belmaker (2026).

Our sampling efforts focused on the continental shelves and upper slopes of two distinct sites: the Northern Red Sea and the Eastern Mediterranean (Figure 2a). In the Red Sea, sampling was conducted in the northern Gulf of Aqaba. This site is characterized by a steep bathymetric slope (Weinstein et al., 2021) and consolidated habitats (e.g. coral and rocky reefs), interspersed with unconsolidated habitats (e.g. gravel, sand and silt). The southern samples are located within the ‘Yam Ha‐Almogim’ marine protected area (UNEP‐WCMC, 2024a), where fishing is prohibited. While the northern samples may experience some fishing activity, it is restricted, and most reef fish species are protected. The sampling site is representative of the typical shelf and upper slope ecosystems across the broader Red Sea. In parallel, in the Eastern Mediterranean, sampling occurred in the Levantine Basin, specifically within and around ‘Yam Rosh Haniqra’ marine protected area (UNEP‐WCMC, 2024b). This site was selected as it contains both consolidated and unconsolidated habitats, unlike other sampling regions southward towards the Suez Canal, and hence is most comparable to the Red Sea. The bathymetry of this region is characterized by a steep slope relative to other Eastern Mediterranean sites, yet the slope is still shallower than in the Red Sea. Offshore fishing is prohibited within this protected area, although the southern sites outside its boundaries are subject to fishing.

### 2.2 Video surveys

Fish assemblages were surveyed using stereo‐BRUVs (Figure 2b; Langlois et al., 2020; Whitmarsh et al., 2017), a non‐extractive approach, especially suitable for protected areas. A complete description of the survey settings and data availability is detailed in Chaikin and Belmaker (2026). As this study utilizes these previously published non‐extractive data, specific licences and permits were not required. The methodology facilitated the estimation of species relative abundance, commonness (occurrences), habitat association and depth distributions. Deployments were conducted on both consolidated and unconsolidated substrates between 5 and 150 m from November 2019 to July 2022. To maximize habitat coverage, a stratified random design with a fixed‐site component was employed, maintaining a minimum horizontal distance of 300 m (average horizontal distance = 693 m) between simultaneous samples. Due to depth differences between simultaneous samples, the actual bathymetric distance a demersal fish must navigate between deployments is inherently greater.

### 2.3 Data processing

The 247 deployed stereo‐BRUVs samples totalled 6806 individuals belonging to 230 species. Yet, to ensure appropriate estimates, we applied data filtering to the raw stereo‐BRUVs dataset. We defined a ‘population’ as the occurrence of a species within a specific basin (Red Sea or Mediterranean Sea). Consequently, species occurring in both basins were treated as two distinct populations for the purpose of paired habitats comparison. From the initial assemblage, we retained populations that could be identified at the species level and excluded singleton observations. This resulted in a final dataset of 179 populations (126 in the Red Sea and 53 in the Mediterranean Sea) representing 170 distinct species suitable for analysis.

To quantify the relative abundance of species per sample, we used the video frame with the maximum number of individuals within a sample (MaxN; cf. Chaikin et al., 2022). This is a conservative measure of relative abundance that eliminates the overestimation of abundance from video surveys (Priede et al., 1994; Whitmarsh et al., 2017). MaxN was particularly suitable as saturated video frames were very rare in our samples (Stobart et al., 2015). Although bait is used to attract and centre fish in front of the cameras, previous studies demonstrate that this method effectively samples a broad range of trophic groups, including herbivores and omnivores (Cheal et al., 2021; French et al., 2021; Harvey et al., 2007). The categorization of species as introduced species was based on a published checklist detailing Red Sea fish records in the Mediterranean Sea (Golani, 2021).

### 2.4 Habitat and demographic metric extraction

We derived a suite of metrics to characterize the habitat use of each population (Figure S1). The commonness of the species was represented by the number of occurrences across all basin‐level samples, while local abundance was captured using mean MaxN. Environmental metrics included the minimum depth, depth range and Sea Bottom Temperature range (SBT range) occupied by the species. While MaxN and commonness are demographic metrics rather than physical habitat features, we included them in our analyses because they are fundamentally intertwined with habitat utilization (i.e. abundant species have a higher probability of being detected across a wider variety of habitats).

To quantify fine‐scale habitat associations, we visually annotated the benthic features present in every stereo‐BRUVs deployment. The standardized habitat categories included: rocky reefs, stony coral reefs (hereafter ‘corals’), sand, silt, seagrass, macroalgae and artificial structures (e.g. large debris, sunken vessels or infrastructure). Because multiple habitat features often co‐occur within a single deployment, all present habitats were recorded. For each species population, habitat associations were then calculated as the proportion of deployments in which a specific habitat feature was present across all the samples where the population appeared. Additionally, habitat breadth was subsequently calculated using Standardized Hurlbert's habitat breadth index (see Section 2.5 below).

To reduce statistical dependence among predictors, we examined multicollinearity using a Pearson correlation matrix (Figure S2). The majority of metric pairs exhibited low correlation (*r* < 0.7), indicating distinct axes of variation. Consequently, we retained the majority of these metrics for the multi‐comparison framework, excluding only specific redundant metrics (see Section 2.6). Intuitively, common species may exhibit greater habitat breadth, allowing them to exploit a wider variety of substrates—characteristics hypothesized to strongly influence invasion success (Table 1). By including species commonness (i.e. sample size) as a covariate, our models partially control for the confounding effect of observation frequency on our habitat estimates.

### 2.5 Habitat breadth

To complement the fine‐scale habitat associations (e.g. corals and sand) described above, we also employed a coarse‐scale habitat classification to calculate the overall habitat breadth of each species. Hurlbert's niche breadth accounts for the possibility that habitats are not equally abundant and thus ideal for the data in hand. For this purpose, we categorized each deployment into one of the three habitats according to the seabed form: *Consolidated* (*a continuous hard substrate, such as a rocky or coral reef, that filled the entire visible frame*), *Unconsolidated* (*a continuous soft substrate, such as gravel, sand or silt*), and *Mixed* (habitats that included both consolidated and unconsolidated substrate patches). We then used Hurlbert's niche breadth (*B′*) to determine the level of habitat breadth of each population (Equation 1), where *P*_*j*_ is the proportion of individuals found in habitat *j*, and *a*_*j*_ is the proportion of the total available samples consisting of habitat *j*. Finally, we standardized Hurlbert's niche breadth (*B*'_*A*_; Equation 2) to a scale of 0–1 where *a*_min_ is the smallest observed proportion of all habitats (Krebs, 1999).

(1) $$B^{\prime }=\frac{1}{\sum \left(\frac{p_{j}^{2}}{a_{j}}\right)}$$

(2) $$B\prime_{A}=\frac{B^{\prime }-a_{\min}}{1-a_{\min}}$$

*B'*_*A*_ is highest when an equal number of individuals occurs in each habitat and thus has the broadest possible habitat breadth.

### 2.6 Statistical analyses

#### 2.6.1 Characterization of the multidimensional habitat space

To characterize the multidimensional ecological space, we first calculated a Gower dissimilarity matrix using the ‘gowdis’ function of the ‘FD’ R package (Laliberté et al., 2023) with a set of variables (Table S2, Figure S1) encompassing habitat associations (e.g. rocks), demography (e.g. MaxN) and environment (e.g. SBT). We visualized ecological separation across assemblages (Red Sea natives, Red Sea Lessepsians, Mediterranean Lessepsians and Mediterranean natives) using Principal Coordinates Analysis (PCoA). To test the distinctiveness between assemblages, we employed Permutational Multivariate Analysis of Variance (PERMANOVA) utilizing ‘adonis2’ (vegan) and ‘pairwise.adonis’ (‘pairwiseAdonis’ package; Martinez Arbizu, 2017) with 9999 permutations. To assess post‐invasion habitat shifts, we performed Procrustes analysis using the ‘protest’ function in the ‘vegan’ R package (9999 permutations), minimizing the sum of squared differences between Red Sea Lessepsians and Mediterranean Sea Lessepsians habitat configurations (Oksanen et al., 2019). Furthermore, taking advantage of the populations occurring in both basins, we conducted a paired analysis within the PCoA space, plotting species‐specific vectors to visualize the direction and magnitude of post‐invasion habitat shifts.

#### 2.6.2 Identifying drivers of assemblage distinctiveness

To identify which specific variables maximized the separation between assemblages, we employed a linear discriminant analysis (LDA) across three comparisons: (1) Native range; (2) Native‐invaded range; and (3) Invaded range (Figure 1). While our PERMANOVA established whether the multivariate space differed between groups, the LDA was employed to pinpoint the variables responsible for driving that separation. In these models, the categorical response variable was the assemblage identity (e.g. native versus introduced species), while the predictors consisted of the continuous demographic, environmental and habitat metrics. To prevent multicollinearity and enhance convergence, predictor variables were dynamically filtered prior to each comparison. Specifically, any variable consisting of zero variance, causing a high Variance Inflation Factor (i.e. VIF >5), or occurring in fewer than five species within a specific assemblage was excluded from that model (Table 2).

**TABLE 2:** Linear discriminant analysis (LDA) loadings separating native and Lessepsian fish assemblages.

Variable	Native‐range comparison	Native‐invaded‐range comparison	Native‐invaded (sensitivity)	Invaded‐range comparison
Algae	−4.695	2.571	−1.892	−0.035
Artificial	0.917	1.352	Sens.	Var.
Commonness	0.008	0.013	−0.013	−0.074
Corals	2.063	5.635	Sens.	Var.
Depth range	−0.009	0.019	0.025	0.018
Habitat breadth	−0.714	−0.897	−1.735	−2.294
MaxN	−0.505	−0.138	0.048	−0.014
Min. depth	0.008	0.017	0.017	0.003
Rocks	0.767	−0.316	−2.474	VIF
Sand	0.894	4.422	−0.909	−1.478
Seagrass	−0.508	5.016	Sens.	Var.
Silt	1.606	VIF	−7.960	VIF
Temperature range	0.077	−0.259	Sens.	−0.065
LOOCV AUC	0.651	0.971	0.827	0.509

*Note*: Values indicate the relative contribution of each variable to the primary discriminant axis. Positive and negative signs reflect the direction of the relationship relative to the group centroids (e.g. in the Native‐range comparison, negative values are associated with Lessepsian species). Variables excluded from specific models prior to analysis are denoted by text tags: VIF (removed due to elevated Variance Inflation Factor >5), Var. (removed due to zero or insufficient variance) and Sens. (removed to restrict the sensitivity analysis to habitats available in both basins). Bottom row denotes the LOOCV AUC of each comparison, informing about the discriminative performance of the models.

To assess the discriminatory performance of these models, we computed out‐of‐sample probabilities directly during model fitting using Leave‐One‐Out Cross‐Validation (LOOCV). We then quantified the out‐of‐sample predictive performance by calculating the Area Under the receiver operating characteristic curve (AUC) using the ‘pROC’ R package (Robin et al., 2011). Alongside evaluating model performance, we determined the relative importance of specific predictors driving the separation by extracting variable loadings from the discriminant functions using the ‘lda’ function in the ‘MASS’ package (Ripley & Venables, 2024). To account for habitat availability differences between the basins during the native‐invaded‐range comparison, we conducted a restricted LDA model, strictly limiting variables to only those that shared similar ranges in both basins (e.g. excluding corals and SBT range). This ensured that the detected separation reflects habitat lability rather than habitat availability.

#### 2.6.3 Predicting native‐range invasion potential

To predict the probability of a species being introduced while controlling for taxonomic relatedness within the native‐range comparison, we fitted a generalized linear mixed model (GLMM) using the ‘lme4’ package (Bates et al., 2015). We used a binomial error distribution with ‘Status’ as the binary response variable (0 = non‐Lessepsian, 1 = Lessepsian). Fixed effects in the global model included 16 scaled continuous variables (MaxN, commonness, habitat breadth, depth range, minimum depth, minimum SBT, maximum SBT and SBT range, artificial structures, corals, seagrass, silt, gravel, sand, rocks and algae), with Family included as a random intercept. To identify the most parsimonious combination of predictors, we evaluated all possible subsets of the global model using the ‘dredge’ function in the ‘MuMIn’ package (Barton, 2023). We selected the best‐performing model based on the lowest Akaike information criterion corrected for small sample sizes (AICc). Finally, we determined the relative importance of each individual predictor by calculating its sum of Akaike weights across all evaluated models. For the selected model, we estimated each fixed effect's relative contribution to the total *R* ^2^m using the ‘glmm.hp’ function of the ‘glmm.hp’ R package (Lai et al., 2022). We verified model diagnostics (Figure S3) using the ‘DHARMa’ package (Hartig, 2021) and assessed multicollinearity through VIF using the ‘performance’ package (Lüdecke et al., 2021).

#### 2.6.4 Ensemble forecasting and risk classification

To establish a robust consensus on potential future invasion candidates, we synthesized the predictions from the LDA and GLMM approaches. We extracted predicted probabilities from both models prior to combination: specifically, the posterior class‐membership probability for the Lessepsian group from the LDA, and the predicted response probability from the GLMM. To reduce model‐specific biases, we combined these probabilities into a performance‐weighted ensemble index, where each model's contribution was weighted by its respective LOOCV AUC score. Species were subsequently ranked based on this ensemble index, and the agreement between the two modelling frameworks was evaluated using Spearman's rank correlation. Finally, to classify high‐priority invasion candidates, we derived an optimal classification threshold by maximizing Youden's J statistic (Youden, 1950), which balances sensitivity and specificity.

## 3 RESULTS

A total of 179 populations (defined as a species occurring within a specific basin) representing 170 unique species met the inclusion criteria and were analysed. The sampled Red Sea assemblage consisted of 108 native populations and 16 Lessepsian populations. In the invaded Mediterranean Sea, the assemblage comprised 34 native populations and 17 Lessepsian populations. To maximize the sample size for cross‐basin habitat shift comparisons, two cosmopolitan species recorded in both basins (*Seriola dumerili* and *Echeneis naucrates*, representing four total populations) were grouped with the Lessepsian assemblages for all downstream analyses.

### 3.1 Habitat and ecological space overview

**FIGURE 3:**
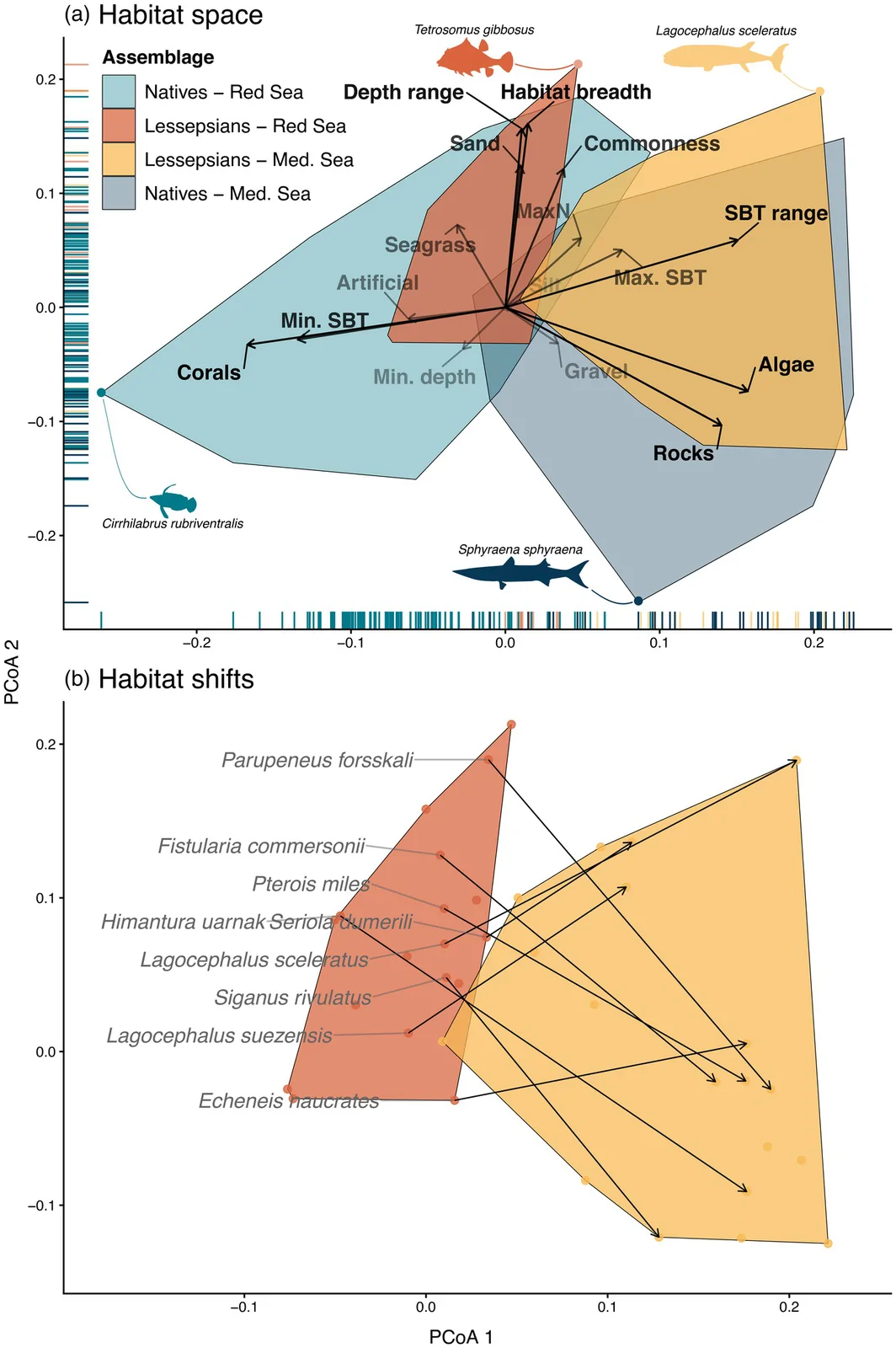
Habitat shifts of native and Lessepsian fish assemblages. (a) Principal Coordinates Analysis (PCoA) illustrating the ecological space of fish populations in the Red Sea and Mediterranean Sea, based on Gower distances of their demographical and habitat metrics. Convex hulls and rug marks delineate the ecological space for native populations (blue shades; Red Sea: *n* = 108 populations, Mediterranean: *n* = 34) and Lessepsian populations (red/orange shades; Red Sea: *n* = 18, Mediterranean: *n* = 19) in each basin. Black vectors indicate the direction and magnitude of variable correlations with the PCoA axes. Silhouettes denote representative candidate species. (b) Habitat shifts of Lessepsian species across both basins (*n* = 18). For shared species that were recorded in both basins, arrows connect the ecological space position of each species in its native range (Red Sea; red) to its invaded range (Mediterranean; orange), representing the magnitude and direction of individual habitat shifts. Longer arrows indicate larger habitat change.

The PCoA revealed distinct ecological structuring among the four assemblages, with the first two axes capturing 59% of the total variation (Figure 3a, Axis 1: 36.1%, Axis 2: 22.9%). A PERMANOVA confirmed that assemblage identity significantly structured this multidimensional space, explaining approximately 30% of the total variation (*F* = 24.8, *R* ^2^ = 0.30, *p* < 0.001). Vectors indicated that the positioning of populations within this space was strongly driven by gradients of rocky habitat (*R* ^2^ = 0.76), macroalgae (*R* ^2^ = 0.75), corals (*R* ^2^ = 0.73), habitat breadth (*R* ^2^ = 0.66), SBT range (*R* ^2^ = 0.66) and depth range (*R* ^2^ = 0.62; Table S2). Specifically, association with corals characterized both the native and Lessepsian populations within the Red Sea. However, Red Sea Lessepsians differentiated themselves from their native counterparts by aligning with vectors denoting larger depth ranges and wider habitat breadths. Conversely, both Mediterranean assemblages (natives and Lessepsians) were primarily characterized by their association with rocky reefs, macroalgae and broader thermal ranges.

### 3.2 Native‐range comparison and forecasting invasion risk

**FIGURE 4:**
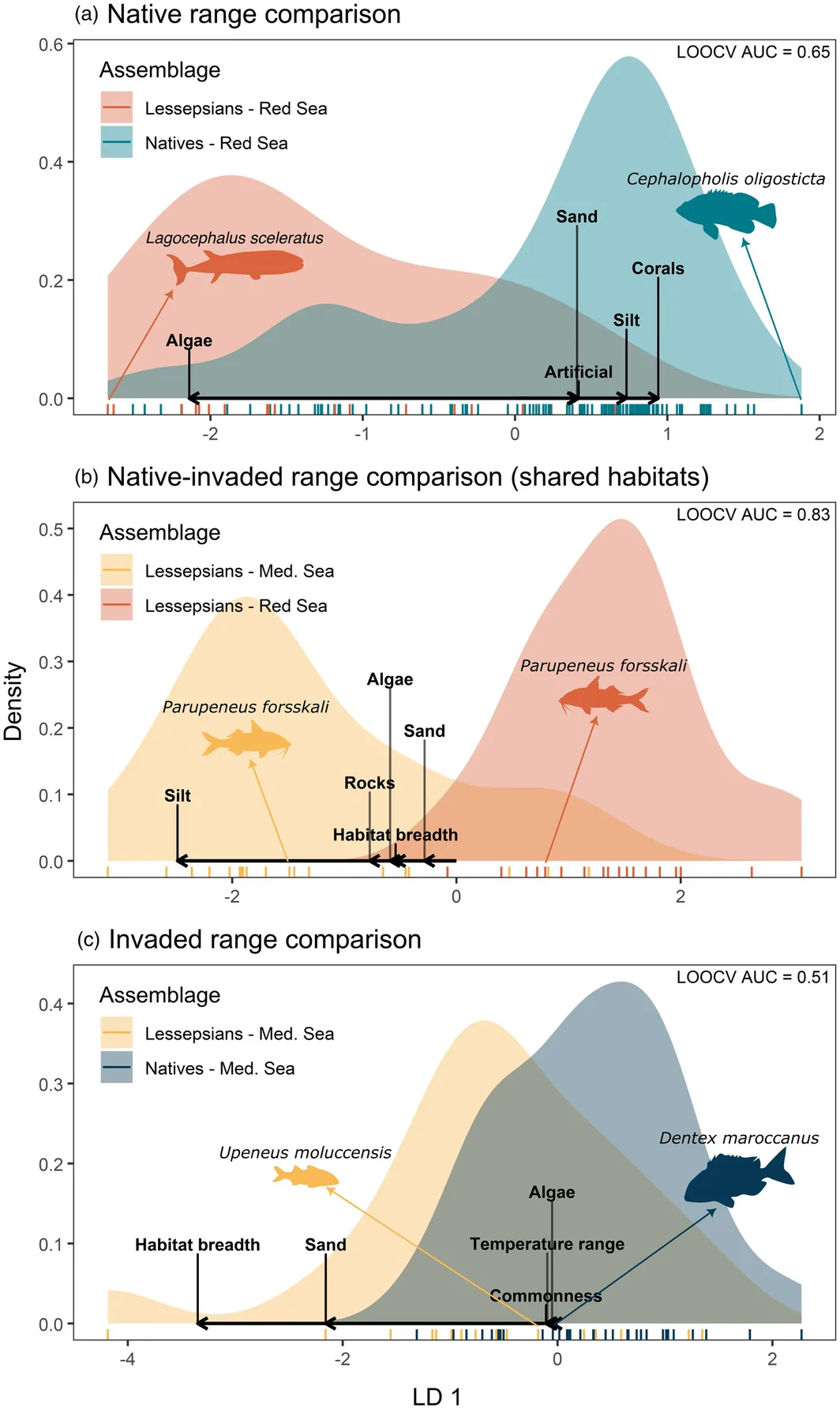
Linear discriminant analysis (LDA) comparing habitat differences for native and Lessepsian fish assemblages. Density plots and rug markers illustrate the distribution of species scores along the linear discriminant axis (LD1) for (a) the native‐range comparison (Red Sea natives: *n* = 108 populations vs. Lessepsians in their native Red Sea range: *n* = 18), identifying habitat affinities associated with invasion potential; (b) the native‐invaded‐range comparison (Lessepsians in the Red Sea: *n* = 18 vs. Lessepsians in the Mediterranean: *n* = 19), specifically restricted to habitats available in both basins to assess active post‐invasion ecological shifts; and (c) the invaded‐range comparison (Lessepsians in the Mediterranean: *n* = 19 vs. Mediterranean natives: *n* = 34), examining establishment in the recipient basin. The LOOCV AUC in each upper‐right corner estimates model classification performance, where a value of 0.50 indicates random classification and 1.00 perfect discrimination. Black arrows denote the direction and magnitude of the five dominant variable loadings driving the separation between assemblages in each model. Fish silhouettes demonstrate candidate species.

**FIGURE 5:**
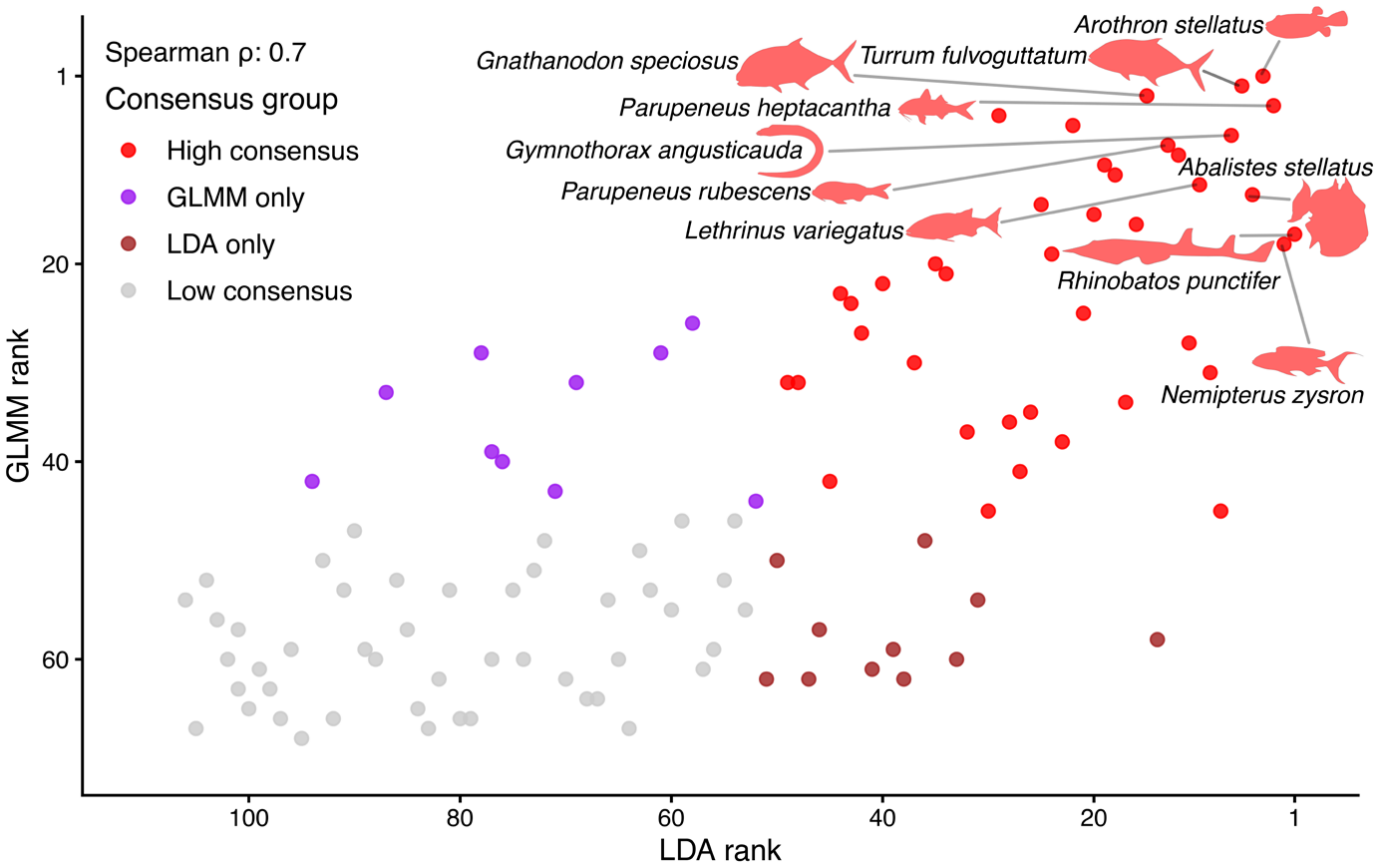
Consensus prediction of invasion potential for Red Sea (non‐introduced) native species. Scatter plot comparing the invasion potential ranks derived from the generalized linear mixed model (GLMM) and linear discriminant analysis (LDA). Ranks (*n* = 108) are reversed so that the highest‐potential species (Rank 1) appear at the top‐right. Points are colour‐coded based on model agreement: ‘High consensus’ (red) indicates species whose predicted probabilities exceed the empirically derived optimal threshold in both models; ‘GLMM only’ (purple) and ‘LDA only’ (brown) indicate probabilities exceeding the threshold in only one model; and ‘Low consensus’ (grey) indicates species falling below the threshold in both models. The 10 highly ranked potential candidates are labelled.

The native‐range comparison yielded a significant ecological separation between non‐introduced species and Lessepsian species (Table 2), with invasion status accounting for 6% of the multivariate ecological variance (Table S3). The LDA discriminated between these groups with an out‐of‐sample LOOCV AUC of 0.651. This separation was primarily driven by substrate associations, with ‘Algae’ loading negative (associated with Lessepsians) and ‘Corals’ loading positive (associated with natives; Figure 4a, Table 2). A complementary GLMM evaluating the introduction potential of the Red Sea non‐introduced species pool identified strong ecological filtering at the source (*R* ^2^m = 0.36, *R* ^2^c = 0.59; Figure S4), achieving a robust out‐of‐sample predictive performance (LOOCV AUC = 0.792). Model selection confirmed that the lack of coral association was the most important predictor of invasion risk (sum of weights = 0.97, Tables S4 and S5), further supported by shallower minimum depths (sum of weights = 0.41). Hierarchical variance partitioning revealed that the lack of coral association accounted for 85.8% of the explained variance. Consensus between the GLMM and LDA rankings was high (Figure 5, Spearman's *ρ* = 0.7, *p* < 0.0001), flagging species such as *Arothron stellatus*, *Parupeneus heptacantha* and *Turrum fulvoguttatum* as high‐risk candidates for future invasion (Table S6). The final performance‐weighted ensemble index combining both frameworks maintained strong overall discriminatory power (LOOCV AUC = 0.756, Figure S5).

### 3.3 Native‐invaded‐range comparison

Comparisons between the native (Red Sea) and invaded (Mediterranean) ranges of Lessepsian species revealed extensive shifts in habitat use. Procrustes analysis indicated a lack of significant structural concordance between the two ranges (*m* ^2^ = 0.911, *r* = 0.298, *p* = 0.615), suggesting that species did not shift uniformly but rather responded individually to the novel Mediterranean environment. This was confirmed by both the PERMANOVA and the LDA, which detected a highly significant ecological separation and yielded the highest out‐of‐sample discriminative performance of the study (PERMANOVA: *R* ^2^ = 0.27, *p* < 0.0001; LDA: LOOCV AUC = 0.971; Table 2, Figure S6, Table S3). The shift was driven almost exclusively by the loss of specific habitat associations; Corals (LD1 = 5.63), Seagrass (LD1 = 5.02) and Sand (LD1 = 4.42) were the strongest drivers separating Red Sea populations from their Mediterranean counterparts (Table 2). To ensure this, separation was not merely caused by the absence of tropical habitats and conditions (e.g. corals, SBT range and seagrass) in the recipient basin, we performed a sensitivity analysis restricting the analytical space strictly to habitats available in both seas (Figure 4b, Table 2). Notably, the models maintained robust discrimination even within this shared habitat space (LOOCV AUC = 0.827). Within this restricted model, the divergence was driven by a reorganization of affinities for the remaining shared habitats—specifically Lessepsians in the Mediterranean showed shifts associated with Silt (LD1 = −7.96), Rocks (LD1 = −2.47) and Algae (LD1 = −1.89)—coupled with positive changes in overall habitat breadth (LD1 = −1.73). In contrast, demographic metrics, such as MaxN (LD1 = 0.048) and commonness (LD1 = −0.013) exhibited negligible discriminatory weight. These results highlight a ‘habitat release’ pattern, where Lessepsians do not just passively lose their tropical habitat affinities, but exhibit flexibility to exploit available Mediterranean habitats with surprisingly little increase in abundance.

### 3.4 Invaded‐range comparison

In the Mediterranean Sea, the habitat associations of Lessepsian species were statistically indistinguishable from the native assemblage (Figure 4c, Table 2, Table S3), and the LDA failed to classify species better than random chance (LOOCV AUC = 0.509). Consequently, no distinct depth or habitat patterns separated Lessepsians from Mediterranean natives (Table 2). While Lessepsians might still partition resources along unmeasured dimensions (e.g. trophic axis), these results indicate that, in terms of habitat use and depth distribution, Lessepsian species have integrated seamlessly into the existing space of the recipient community rather than occupying a specific habitat or depth.

## 4 DISCUSSION

To effectively predict which species will become introduced, it is critical to separate intrinsic pre‐adaptations from post‐invasion ecological shifts (van Kleunen et al., 2010). By deploying a comprehensive three‐comparison framework using standardized in situ data across the Red Sea and Mediterranean Sea, we decoupled these processes. Contrary to invasion hypotheses that rely on ecological generalism or high propagule pressure via abundance, our findings reveal that an important source‐pool filter for Lessepsian species is a specific habitat pre‐adaptation: a distinct lack of reliance on coral habitats. Furthermore, we demonstrate that establishment is characterized by post‐invasion habitat lability and an overall integration into the recipient community that contrasts with previous suggestions of filling vacant niches.

Our native‐range comparisons provide strong evidence for ecological filtering at the source. The strongest predictor of invasion potential within the Red Sea pool was not being a generalist, but specifically exhibiting independence from coral substrates and a positive association with macroalgae. This acts as a pronounced environmental filter, as the Suez Canal and the Mediterranean Sea largely lack the complex, reef‐building coral habitats that characterize the Red Sea (Stanley & Goodfriend, 1997). Consequently, species strictly dependent on corals are suggested to be filtered out early in the invasion process. Introduced species are thus those pre‐adapted to persist in the absence of reefs that they probably do not encounter during transit and upon arrival. These quantitative findings offer a refined, empirical update to early observations (Golani, 1993). While Golani originally posited that unconsolidated environments—specifically the sandy shores of the Red Sea—acted as the primary ‘launching pad’ for Lessepsian species, our data indicate that a specific affinity for sand is not the primary statistical driver. Rather, it is the fundamental independence from coral that facilitates the pool of species pre‐adapted to survive the reefless expanses of the Suez Canal and the Mediterranean Sea.

While independence from coral substrates acts as the primary filter for invasion, our analyses indicate that generalism still holds an important, albeit subordinate, role in shaping invasion potential. In the unconstrained ecological space (Figure 3a, Table S2), vectors for depth range and habitat breadth constituted major axes of variation with Lessepsian species consistently aligning towards the generalist spectrum. This aligns with our multi‐model inference, where depth range exhibited a positive effect on invasion status. Importantly, this broad depth range effect was coupled with the filtering effect of shallower minimum depths, supporting the prediction that species already adapted to persist in shallow environments are inherently more likely to enter this corridor (Table 1). These findings provide empirical support for the ‘niche breadth hypothesis’ (Hutchinson, 1957; Levins, 1968), where generalists are inherently equipped to expand into novel environments (Higgins & Richardson, 2014; Slatyer et al., 2013). However, the discriminatory weight of specific habitat affinities in our models highlights a nuance in Lessepsian migration: ecological generalism is advantageous, but it is fundamentally secondary to the environmental filter associated with being independent of coral habitat. Ultimately, a species with a broad depth range may not successfully migrate if it is obligately bound to reef‐building corals.

By understanding these invasion mechanisms, we can move from observation to proactive forecasting. By synthesizing linear discriminant and mixed‐modelling approaches into a unified ensemble index, we ranked the remaining observed Red Sea native pool for their invasion potential (Table S6, Figure 5). This consensus approach minimizes biases of individual statistical models while controlling for taxonomic relatedness. The models flagged specific species that are less reliant on corals, such as *Parupeneus heptacantha*, *Arothron stellatus* and *Turrum fulvoguttatum*, as high‐potential candidates for future Mediterranean establishment. As the Mediterranean continues to warm and tropicalize (Chust et al., 2024), generating and refining these watchlists provides a critical tool for local management and early detection efforts.

While native‐range affinities dictate which species have the potential to invade, our cross‐basin comparisons reveal that successful establishment is accompanied by ecological lability rather than strict habitat conservatism. By tracking the whole Lessepsian assemblages, as well as specifically the shared Lessepsians species, across the basins we observed a pattern of habitat release wherein introduced species abandon their native habitat associations to exploit available Mediterranean habitats (Figure 3b). Although empirical comparisons for marine fishes remain exceedingly rare, our findings align with emerging evidence from specific cases (Riley et al., 2014). This lability challenges the paradigm of habitat conservatism widely documented in broad meta‐analyses of terrestrial plants (Hejda et al., 2015) and diverges from other Lessepsian taxa, such as ascidians, suggested to conserve their native habitat use patterns (Granot et al., 2017).

Crucially, we found that this post‐invasion lability is mostly restricted to habitat use, whereas demographic characteristics—such as abundance and commonness—remained notably stable across basins. Surprisingly, our data provided no evidence that introduced species undergo fundamental demographic shifts to become superabundant in the invaded range. While there are undeniable examples of introduced species (Côté et al., 2013; Sala et al., 2011) or range‐expanding species (Jepsen et al., 2008; Ling et al., 2009) achieving superabundance and dominating recipient ecosystems, this pattern did not emerge as a general rule within our study. This stability challenges the common perception that introduced species are inherently hyper‐prolific in the invaded range. Instead, it indicates that many introduced species integrate into novel environments by successfully shifting the habitats they establish, without undergoing profound shifts in their population dynamics (Golani, 1998; Williamson & Fitter, 1996a).

Once established, Lessepsian species were assimilated into the Mediterranean ecosystem, becoming indistinguishable from the native community in terms of habitat use (with models failing to classify them better than random chance). While earlier literature focusing on species‐level ecological and morphological traits indicates that introduced species often fill vacant niches with little overlap with natives (Arndt et al., 2018; Azzurro et al., 2014; Givan et al., 2017), our study adds the dimension of fine‐scale habitat use. Interestingly, along this specific niche axis, we observe the opposite pattern, with Lessepsian species exhibiting substantial overlap with the native community. In addition, our methodology relies on in situ realized population‐level habitat use rather than coarse, species‐level potential. By measuring how fish actually behave and utilize habitats locally, we may capture a different picture of their niche use. Consequently, habitat use overlap suggests an elevated potential for direct competition between native and Lessepsian species within the warming Mediterranean ecosystem. At the same time, this approach does not capture the ecological overlap between Lessepsian species and natives in other factors, such as diet (Arndt et al., 2018; Golani, 1994).

Five main caveats warrant consideration when interpreting our findings. First, our sampling was geographically restricted within each basin, yet it successfully encompassed highly diverse habitats down to depths of 150 m. This environmental coverage is ecologically representative, capturing the unconsolidated nature of the Suez Canal pathway while allowing direct comparisons with consolidated habitats further north in the Mediterranean. Second, relying on visual census introduces inherent limitations, such as difficulties in distinguishing morphologically similar congeners (*Fistularia commersonii* and *F. petimba*). However, because such congeners often share invasion status and ecology, this is unlikely to systematically skew habitat‐use predictions. Third, estimating in situ habitat affinities for rare species inherently carries higher uncertainty, as low observation counts can underestimate habitat breadth. However, we mitigated this limitation by excluding singleton observations from the dataset and explicitly incorporating species commonness as a covariate in our mixed‐modelling framework. Fourth, targeted fisheries in the Mediterranean can deplete native predators (e.g. groupers), reducing biotic resistance and expanding the availability of vacant niches for opportunistic Lessepsian species (Giakoumi et al., 2018). Reduced resistance may lower the overall barrier to entry, yet combine with species' intrinsic traits to dictate its establishment success (Belmaker et al., 2013; Shea & Chesson, 2002). Fifth, while our GLMM partially addresses species non‐independence using family as random intercept, it may not fully account for deeper residual phylogenetic structure (Johnson et al., 2024). However, the strong consensus between this model and our cross‐validated LDA framework (which evaluates empirical out‐of‐sample predictive performance rather than relying solely on in‐sample inference) provides confidence that the identified ecological drivers are of ecological importance.

We note that Lessepsian migration represents a complex intersection of human‐assisted introduction (via the Suez Canal) and climate‐driven range expansion (Chust et al., 2024; Schickele et al., 2021). Consequently, it can be ambiguous to determine which process dominates our observations. Nevertheless, rather than being a limitation, this duality captures the ‘global bioflow’ (Carlton & Schwindt, 2026), highlighting the potential for knowledge transfer between invasion biology and climate change ecology. This suggests that the mechanisms identified here—specific source‐pool pre‐adaptations and post‐expansion niche lability—are also relevant for modelling global climate‐driven range shifts (Hayes et al., 2024; Sorte et al., 2010; Stuart‐Smith et al., 2021; Wallingford et al., 2020).

## 5 CONCLUSIONS

To accurately predict marine invasions, ecological assessments must go beyond the invaded range and evaluate the entire invasion pathway. In a comprehensive, in situ comparative analysis, we demonstrate that fish invasions in this system rely on a two‐stage ecological process. First, initial invasion potential is heavily gated by a specific, dominant source‐pool pre‐adaptation: the distinct independence from coral habitats that may allow species to overcome the environmental filter of the Suez Canal. Second, once this geographical barrier is breached, establishment in the novel environment is subjected to habitat lability. Consequently, this habitat lability means that harnessing habitat use in the native range to predict expansion potential in the invaded range may lead to serious underestimations of invasion range size or impact. As ocean warming continues to accelerate the redistribution of marine species, integrating both native‐range habitat pre‐adaptations and post‐invasion ecological dynamics into predictive models will be paramount for anticipating and managing future shifts.

## AUTHOR CONTRIBUTIONS

Shahar Chaikin and Jonathan Belmaker conceived the ideas; Shahar Chaikin, Shahar Malamud and Tal Gavriel collected the data. Shahar Chaikin led the formal analysis and visualization. Jonathan Belmaker supervised the study. Shahar Chaikin and Jonathan Belmaker led the writing of the original manuscript with writing support from Avery Deveto. All authors contributed to the drafts.

## CONFLICT OF INTEREST STATEMENT

The authors declare that this study was conducted without any commercial or financial relationship that could be construed as a potential conflict of interest.

## Supporting information


**Figure S1.** Distribution of variables across the four studied fish assemblages. Grey points represent the raw variable values for individual populations. Solid coloured points and associated error bars indicate the mean and 95% confidence intervals for each assemblage, respectively. To reflect the invasion pathway, assemblages are ordered sequentially from left to right: non‐introduced species in the source pool (Natives—Red Sea), successful introduced species evaluated in their native range (Lessepsians—Red Sea), successful introduced species evaluated in the recipient range (Lessepsians—Med. Sea), and the native community in the invaded range (Natives—Med. Sea). Note that the y‐axes are scaled independently for each panel to accommodate different units of measurement.
**Figure S2.** Pearson correlation matrix of variables investigated in this study before their inclusion in the analyses. The colour gradient indicates the strength and direction of the correlation, ranging from negative (blue) to positive (red).
**Figure S3.** Diagnostic plots of the simulated residuals for the GLMM evaluating the probability of Red Sea natives becoming Lessepsian migrants. Residuals were randomized and calculated using the ‘DHARMa’ R package. (Left) A QQ plot of the standardized quantile residuals comparing the expected uniform distribution against the observed residuals, testing for overall distributional assumptions (Kolmogorov–Smirnov test), overdispersion and the presence of outliers. (Right) Simulated residuals plotted against the model's predicted values to visually assess homoscedasticity and functional form. The horizontal quantile regression lines (red) indicate that the residuals are evenly distributed, confirming that the model assumptions are met without deviations.
**Figure S4.** Determinants of invasion potential among Red Sea fish species. Standardized fixed‐effect coefficients (95% confidence intervals) from a GLMM distinguishing between Red Sea natives and successful Lessepsian migrants. Positive coefficients indicate variables associated with a higher probability of being a migrant (i.e. higher invasion potential). Association with corals (red) was the only significant predictor, while grey error bars indicate non‐significant terms overlapping the zero line. Values appended to the *y*‐axis labels denote the unique variance explained by each predictor (marginal *R* ^2^ and its relative percentage) derived via hierarchical variance partitioning. Overall model variance is indicated by marginal (*R* ^2^m; variance explained by fixed effects) and conditional (*R* ^2^c; variance explained by the entire model) coefficients of determination.
**Figure S5.** ROC curve evaluating the out‐of‐sample classification of the performance‐weighted ensemble model. The solid blue line represents the cross‐validated ability of the ensemble index to discriminate between Red Sea natives and successful Lessepsian migrants. The dashed diagonal line indicates the baseline of random classification (AUC = 0.5). The red point highlights the empirically derived optimal risk threshold (0.032), which was calculated by maximizing Youden's J statistic to objectively balance sensitivity (true positive rate) and specificity (true negative rate). The overall discriminatory power of the ensemble model is denoted by the LOOCV AUC (0.756).
**Figure S6.** LDA comparing habitat differences for Lessepsian fish assemblages. Density plots and rug markers illustrate the distribution of species scores along the linear discriminant axis (LD1) for the native‐invaded range comparison (Lessepsians in the Red Sea: *n* = 18 vs. Lessepsians in the Mediterranean: *n* = 19). The LOOCV AUC in the upper‐right corner estimates model classification performance, where a value of 0.50 indicates random classification and 1.00 perfect discrimination. Black arrows denote the direction and magnitude of the five dominant variable loadings driving the separation between assemblages in each model.
**Table S1.** Standardized glossary following contemporary definitions in biological invasions (Carlton & Schwindt, 2026).
**Table S2.** Results of the vector fitting analysis (envfit) correlating habitat affinities with the first two axes of the Principal Coordinates Analysis (PCoA). Columns indicate the direction cosines (PCoA1 and PCoA2) representing the orientation of the variable gradient, the *R* ^2^ represents the strength of the association, and the statistical significance derived from 999 permutations. Variables are ordered by the *R* ^2^.
**Table S3.** Pairwise PERMANOVA testing differences in the multidimensional space of native and Lessepsian fish assemblages. *R* ^2^ values indicate the proportion of multivariate variance explained by assemblage identity. All cross‐basin and native‐range comparisons revealed highly significant ecological separation. Notably, the invaded‐range comparison yielded no significant difference.
**Table S4.** Model selection table showing the best 10 model combinations out of 65,536 models.
**Table S5.** Relative importance of predictors based on the sum of AIC weights (SW). The SW for a predictor is the sum of the AIC weights of all models in the candidate set that contain that variable. It ranges from 0 to 1 and represents the evidence for a variable's inclusion in the most probable set of models (i.e. a predictor with a SW of 1 is highly important as it appears in all the best‐supported models).
**Table S6.** Ensemble assessment of potential future Lessepsian migrants. The table ranks native Red Sea species based on their predicted probability of successfully establishing in the Mediterranean Sea. Ranks are provided for each individual model and the performance‐weighted combined ensemble. Species are categorized by their consensus group: ‘High consensus’ indicates species whose predicted probabilities exceed the empirically derived optimal threshold in both independent models; ‘LDA only’ and ‘GLMM only’ indicate species exceeding the threshold (Youden's J = 0.032) in only one framework; and ‘Low consensus’ indicates species falling below the threshold in both models.

## Data Availability

The data supporting the findings of this study are openly available on Zenodo at https://zenodo.org/records/18097061 (Chaikin & Belmaker, 2026). The code used to produce the results is publicly accessible on GitHub at https://github.com/shahar710/JAE‐2025‐01071.git.
