# Supplementary material for: Native habitat affinities predict fish invasions with post‐invasion habitat shifts
Source: J Anim Ecol. 2026 Jul 1;95(9):1762–77. doi: 10.1111/1365-2656.70293 (PMC13536195; doi:10.1111/1365-2656.70293)
Supplement: Supplementary file 1 [file JANE-95-1762-s001.docx]

# Supplemental Figures

**Figure S1. Distribution of variables across the four studied fish assemblages.** Grey points represent the raw variable values for individual populations. Solid colored points and associated error bars indicate the mean and 95% confidence intervals for each assemblage, respectively. To reflect the invasion pathway, assemblages are ordered sequentially from left to right: non-introduced species in the source pool (Natives - Red Sea), successful introduced species evaluated in their native range (Lessepsians - Red Sea), successful introduced species evaluated in the recipient range (Lessepsians - Med. Sea), and the native community in the invaded range (Natives - Med. Sea). Note that the y-axes are scaled independently for each panel to accommodate different units of measurement.

**Figure S2. Pearson correlation matrix of variables investigated in this study before their inclusion in the analyses.** The color gradient indicates the strength and direction of the correlation, ranging from negative (blue) to positive (red).

**Figure S3. Diagnostic plots of the simulated residuals for the GLMM evaluating the probability of Red Sea natives becoming Lessepsian migrants**. Residuals were randomized and calculated using the ‘DHARMa’ R package. (Left) A QQ plot of the standardized quantile residuals comparing the expected uniform distribution against the observed residuals, testing for overall distributional assumptions (Kolmogorov-Smirnov test), overdispersion, and the presence of outliers. (Right) Simulated residuals plotted against the model's predicted values to visually assess homoscedasticity and functional form. The horizontal quantile regression lines (red) indicate that the residuals are evenly distributed, confirming that the model assumptions are met without deviations.

**Figure S4. Determinants of invasion potential among Red Sea fish species.** Standardized fixed-effect coefficients (95% confidence intervals) from a GLMM distinguishing between Red Sea natives and successful Lessepsian migrants. Positive coefficients indicate variables associated with a higher probability of being a migrant (i.e., higher invasion potential). Association with corals (red) was the only significant predictor, while grey error bars indicate non-significant terms overlapping the zero line. Values appended to the y-axis labels denote the unique variance explained by each predictor (marginal R² and its relative percentage) derived via hierarchical variance partitioning. Overall model variance is indicated by marginal (R²m; variance explained by fixed effects) and conditional (R²c; variance explained by the entire model) coefficients of determination.

**Figure S5. ROC curve evaluating the out-of-sample classification of the performance-weighted ensemble model.** The solid blue line represents the cross-validated ability of the ensemble index to discriminate between Red Sea natives and successful Lessepsian migrants. The dashed diagonal line indicates the baseline of random classification (AUC = 0.5). The red point highlights the empirically derived optimal risk threshold (0.032), which was calculated by maximizing Youden’s J statistic to objectively balance sensitivity (true positive rate) and specificity (true negative rate). The overall discriminatory power of the ensemble model is denoted by the LOOCV AUC (0.756).

**Figure S6. LDA comparing habitat differences for Lessepsian fish assemblages.** Density plots and rug markers illustrate the distribution of species scores along the linear discriminant axis (LD1) for the native-invaded range comparison (Lessepsians in the Red Sea: n = 18 vs. Lessepsians in the Mediterranean: n = 19). The LOOCV AUC in the upper-right corner estimates model classification performance, where a value of 0.50 indicates random classification and 1.00 perfect discrimination. Black arrows denote the direction and magnitude of the five dominant variable loadings driving the separation between assemblages in each model.

# Supplemental Tables

**Table S1. Standardized glossary following contemporary definitions in biological invasions** [**(Carlton & Schwindt 2026)**](https://sciwheel.com/work/citation?ids=18609421&pre=&suf=&sa=0)**.**

| **Term** | **Definition** |
| --- | --- |
| Biological Invasion | The overarching process of a species arriving in a novel territory, regardless of whether it is human-mediated or based on climate tracking. |
| Introduced species | Any species that has expanded its range strictly through human mediation or anthropogenic corridors (e.g., the Suez Canal). |
| Range-expanding species | Any species expanding its geographical range naturally by tracking environmental shifts or its climatic niche. |
| Lessepsian species | A geographically specific subset of introduced species, strictly referring to the unidirectional influx of Red Sea biota into the Mediterranean Sea via the Suez Canal |
| Native species | Species occurring strictly within their historically documented geographic range. |
| Non-introduced species | An operational term used to emphasize species occurring in the native range (e.g., the Red Sea) alongside introduced species, but which specifically belong to the pool that has not been introduced elsewhere. |

**Table S2. Results of the vector fitting analysis (envfit) correlating habitat affinities with the first two axes of the Principal Coordinates Analysis (PCoA)**. Columns indicate the direction cosines (PCoA1, PCoA2) representing the orientation of the variable gradient, the R^2^ represents the strength of the association, and the statistical significance derived from 999 permutations. Variables are ordered by the R^2^.

| **Variable** | **PCoA1** | **PCoA2** | **R^2^** | **P value** |
| --- | --- | --- | --- | --- |
| Rocks | 0.700 | -0.518 | 0.759 | 0.001 |
| Algae | 0.786 | -0.368 | 0.752 | 0.001 |
| Corals | -0.836 | -0.164 | 0.726 | 0.001 |
| SBT range | 0.754 | 0.295 | 0.655 | 0.001 |
| Habitat breadth | 0.073 | 0.806 | 0.655 | 0.001 |
| Depth range | 0.053 | 0.783 | 0.616 | 0.001 |
| Min. SBT | -0.673 | -0.141 | 0.472 | 0.001 |
| Commonness | 0.189 | 0.607 | 0.405 | 0.001 |
| Sand | 0.050 | 0.621 | 0.389 | 0.001 |
| Max. SBT | 0.378 | 0.252 | 0.206 | 0.001 |
| Seagrass | -0.156 | 0.362 | 0.156 | 0.001 |
| MaxN | 0.245 | 0.304 | 0.153 | 0.001 |
| Artificial | -0.315 | -0.050 | 0.101 | 0.001 |
| Min. depth | -0.138 | -0.183 | 0.053 | 0.009 |
| Gravel | 0.171 | -0.159 | 0.054 | 0.011 |
| Silt | 0.010 | -0.016 | 0.000 | 0.967 |

**Table S3. Pairwise PERMANOVA testing differences in the multidimensional space of native and Lessepsian fish assemblages.** R^2^ values indicate the proportion of multivariate variance explained by assemblage identity. All cross-basin and native-range comparisons revealed highly significant ecological separation. Notably, the invaded-range comparison yielded no significant difference.

| **Pairs** | **Sum of squares** | **F value** | **R^2^** | **P value** |
| --- | --- | --- | --- | --- |
| Natives - Red Sea vs Natives - Med. Sea | 1.1246 | 54.7026 | 0.28 | 0.0001 |
| Natives - Red Sea vs Lessepsians - Red Sea | 0.1472 | 8.5998 | 0.06 | 0.0001 |
| Natives - Red Sea vs Lessepsians - Med. Sea | 0.6808 | 37.0393 | 0.23 | 0.0001 |
| Natives - Med. Sea vs Lessepsians - Red Sea | 0.4364 | 16.6751 | 0.25 | 0.0001 |
| Natives - Med. Sea vs Lessepsians - Med. Sea | 0.0395 | 1.3582 | 0.03 | 0.2481 |
| Lessepsians - Red Sea vs Lessepsians - Med. Sea | 0.2634 | 12.6688 | 0.27 | 0.0001 |

**Table S4. Model selection table showing the best 10 model combinations out of 65,536 models.**

| Model | Artificial | Habitat breadth | Corals | Depth range | Gravel | Algae | MaxN | Min. depth | Commonness | Rocks | Sand | Max. SBT | Min. SBT | SBT range | Seagrass | Silt | df | logLik | AICc | delta | weight |
| --- | --- | --- | --- | --- | --- | --- | --- | --- | --- | --- | --- | --- | --- | --- | --- | --- | --- | --- | --- | --- | --- |
| 1 | - | - | -1.528 | - | - | - | - | -0.785 | - | - | - | - | - | - | - | - | 4 | -39.930 | 88.191 | 0.000 | 0.003 |
| 2 | - | - | -1.529 | - | - | - | - | - | - | - | - | - | - | - | - | - | 3 | -41.001 | 88.199 | 0.008 | 0.003 |
| 3 | - | - | -1.720 | - | - | - | 0.480 | - | - | - | - | - | - | - | - | - | 4 | -39.981 | 88.292 | 0.101 | 0.003 |
| 4 | - | - | -1.282 | - | - | 0.400 | - | -1.103 | - | - | - | - | - | - | - | - | 5 | -38.938 | 88.376 | 0.185 | 0.002 |
| 5 | - | - | -1.402 | 0.434 | - | - | - | - | - | - | - | - | - | - | - | - | 4 | -40.150 | 88.630 | 0.439 | 0.002 |
| 6 | - | - | -1.406 | 0.402 | - | - | - | -0.746 | - | - | - | - | - | - | - | - | 5 | -39.205 | 88.909 | 0.718 | 0.002 |
| 7 | - | - | -1.630 | - | - | - | 0.378 | -0.669 | - | - | - | - | - | - | - | - | 5 | -39.243 | 88.986 | 0.795 | 0.002 |
| 8 | - | - | -1.244 | 0.589 | - | - | - | - | - | - | - | - | - | - | - | -0.749 | 5 | -39.244 | 88.987 | 0.796 | 0.002 |
| 9 | - | - | -1.471 | - | - | - | - | - | - | - | - | - | - | 0.363 | - | - | 4 | -40.414 | 89.159 | 0.968 | 0.002 |
| 10 | - | - | -1.537 | - | - | - | - | - | 0.290 | - | - | - | - | - | - | - | 4 | -40.428 | 89.187 | 0.996 | 0.002 |

**Table S5. Relative importance of predictors based on the sum of AIC weights (SW).** The SW for a predictor is the sum of the AIC weights of all models in the candidate set that contain that variable. It ranges from 0 to 1 and represents the evidence for a variable's inclusion in the most probable set of models (i.e., a predictor with a SW of 1 is highly important as it appears in all the best-supported models).

| **Predictor** | **Sum of weights** |
| --- | --- |
| Corals | 0.965 |
| Min. depth | 0.409 |
| Algae | 0.360 |
| MaxN | 0.354 |
| Depth range | 0.354 |
| Silt | 0.329 |
| Habitat breadth | 0.327 |
| Sand | 0.290 |
| Min. SBT | 0.285 |
| Rocks | 0.281 |
| Seagrass | 0.264 |
| Commonness | 0.264 |
| Gravel | 0.258 |
| Artificial | 0.258 |
| SBT range | 0.238 |
| Max. SBT | 0 |

**Table S6. Ensemble assessment of potential future Lessepsian migrants.** The table ranks native Red Sea species based on their predicted probability of successfully establishing in the Mediterranean Sea. Ranks are provided for each individual model and the performance-weighted combined ensemble. Species are categorized by their consensus group: “High consensus” indicates species whose predicted probabilities exceed the empirically derived optimal threshold in both independent models; “LDA only” and “GLMM only” indicate species exceeding the threshold (Youden's J = 0.032) in only one framework; and “Low consensus” indicates species falling below the threshold in both models.

| **#** | **Species** | **Family** | **GLMM probs.** | **LDA probs.** | **Ensemble probs.** | **Consensus group** |
| --- | --- | --- | --- | --- | --- | --- |
| 1 | *Arothron stellatus* | Tetraodontidae | 0.52 | 0.67 | 0.59 | High consensus |
| 2 | *Parupeneus heptacantha* | Mullidae | 0.41 | 0.71 | 0.55 | High consensus |
| 3 | *Turrum fulvoguttatum* | Carangidae | 0.50 | 0.50 | 0.50 | High consensus |
| 4 | *Rhinobatos punctifer* | Rhinobatidae | 0.21 | 0.76 | 0.46 | High consensus |
| 5 | *Nemipterus zysron* | Nemipteridae | 0.16 | 0.74 | 0.42 | High consensus |
| 6 | *Abalistes stellatus* | Balistidae | 0.26 | 0.56 | 0.39 | High consensus |
| 7 | *Gnathanodon speciosus* | Carangidae | 0.44 | 0.32 | 0.39 | High consensus |
| 8 | *Gymnothorax angusticauda* | Muraenidae | 0.31 | 0.45 | 0.38 | High consensus |
| 9 | *Lethrinus variegatus* | Lethrinidae | 0.27 | 0.39 | 0.32 | High consensus |
| 10 | *Parupeneus rubescens* | Mullidae | 0.30 | 0.34 | 0.32 | High consensus |
| 11 | *Amphiprion bicinctus* | Pomacentridae | 0.29 | 0.34 | 0.31 | High consensus |
| 12 | *Dascyllus trimaculatus* | Pomacentridae | 0.28 | 0.29 | 0.29 | High consensus |
| 13 | *Arothron hispidus* | Tetraodontidae | 0.29 | 0.27 | 0.28 | High consensus |
| 14 | *Heniochus diphreutes* | Chaetodontidae | 0.41 | 0.11 | 0.27 | High consensus |
| 15 | *Scolopsis ghanam* | Nemipteridae | 0.33 | 0.19 | 0.27 | High consensus |
| 16 | *Gymnothorax griseus* | Muraenidae | 0.22 | 0.31 | 0.26 | High consensus |
| 17 | *Gymnocranius grandoculis* | Lethrinidae | 0.09 | 0.41 | 0.23 | High consensus |
| 18 | *Lethrinus xanthochilus* | Lethrinidae | 0.22 | 0.24 | 0.23 | High consensus |
| 19 | *Oxycheilinus mentalis* | Labridae | 0.10 | 0.35 | 0.21 | High consensus |
| 20 | *Lethrinus borbonicus* | Lethrinidae | 0.03 | 0.43 | 0.21 | High consensus |
| 21 | *Canthigaster cyanospilota* | Tetraodontidae | 0.23 | 0.12 | 0.18 | High consensus |
| 22 | *Lethrinus nebulosus* | Lethrinidae | 0.07 | 0.30 | 0.17 | High consensus |
| 23 | *Paracheilinus octotaenia* | Labridae | 0.01 | 0.33 | 0.16 | LDA only |
| 24 | *Meiacanthus nigrolineatus* | Blenniidae | 0.12 | 0.20 | 0.15 | High consensus |
| 25 | *Amblyeleotris sungami* | Gobiidae | 0.16 | 0.15 | 0.15 | High consensus |
| 26 | *Myrichthys maculosus* | Ophichthidae | 0.14 | 0.06 | 0.10 | High consensus |
| 27 | *Ostracion cyanurus* | Ostraciidae | 0.14 | 0.06 | 0.10 | High consensus |
| 28 | *Choerodon robustus* | Labridae | 0.06 | 0.16 | 0.10 | High consensus |
| 29 | *Variola louti* | Serranidae | 0.14 | 0.05 | 0.10 | High consensus |
| 30 | *Chaetodon paucifasciatus* | Chaetodontidae | 0.14 | 0.04 | 0.09 | High consensus |
| 31 | *Cephalopholis argus* | Serranidae | 0.14 | 0.04 | 0.09 | High consensus |
| 32 | *Labroides dimidiatus* | Labridae | 0.06 | 0.12 | 0.09 | High consensus |
| 33 | *Scarus fuscopurpureus* | Scaridae | 0.06 | 0.11 | 0.08 | High consensus |
| 34 | *Parupeneus macronemus* | Mullidae | 0.09 | 0.06 | 0.08 | High consensus |
| 35 | *Naso unicornis* | Acanthuridae | 0.05 | 0.11 | 0.08 | High consensus |
| 36 | *Pseudanthias taeniatus* | Serranidae | 0.10 | 0.04 | 0.08 | High consensus |
| 37 | *Chaetodon auriga* | Chaetodontidae | 0.11 | 0.03 | 0.07 | GLMM only |
| 38 | *Chaetodon fasciatus* | Chaetodontidae | 0.10 | 0.03 | 0.07 | GLMM only |
| 39 | *Cephalopholis miniata* | Serranidae | 0.09 | 0.04 | 0.06 | High consensus |
| 40 | *Chaetodon melannotus* | Chaetodontidae | 0.10 | 0.02 | 0.06 | GLMM only |
| 41 | *Pseudanthias squamipinnis* | Serranidae | 0.09 | 0.04 | 0.06 | High consensus |
| 42 | *Apolemichthys xanthotis* | Pomacanthidae | 0.06 | 0.07 | 0.06 | High consensus |
| 43 | *Coris aygula* | Labridae | 0.03 | 0.10 | 0.06 | High consensus |
| 44 | *Plectropomus pessuliferus* | Serranidae | 0.09 | 0.02 | 0.06 | GLMM only |
| 45 | *Cephalopholis hemistiktos* | Serranidae | 0.08 | 0.02 | 0.05 | GLMM only |
| 46 | *Gymnothorax johnsoni* | Muraenidae | 0.02 | 0.07 | 0.04 | LDA only |
| 47 | *Anampses lineatus* | Labridae | 0.04 | 0.04 | 0.04 | High consensus |
| 48 | *Scarus ferrugineus* | Scaridae | 0.03 | 0.06 | 0.04 | LDA only |
| 49 | *Chaetodon lineolatus* | Chaetodontidae | 0.05 | 0.02 | 0.04 | GLMM only |
| 50 | *Siganus argenteus* | Siganidae | 0.05 | 0.02 | 0.04 | GLMM only |
| 51 | *Gymnothorax nudivomer* | Muraenidae | 0.01 | 0.06 | 0.04 | LDA only |
| 52 | *Argyrops spinifer* | Sparidae | 0.04 | 0.03 | 0.03 | GLMM only |
| 53 | *Bodianus anthioides* | Labridae | 0.01 | 0.05 | 0.03 | LDA only |
| 54 | *Parupeneus cyclostomus* | Mullidae | 0.04 | 0.02 | 0.03 | GLMM only |
| 55 | *Pseudobalistes fuscus* | Balistidae | 0.03 | 0.03 | 0.03 | Low consensus |
| 56 | *Genicanthus caudovittatus* | Pomacanthidae | 0.02 | 0.04 | 0.03 | LDA only |
| 57 | *Cirrhilabrus rubriventralis* | Labridae | 0.01 | 0.05 | 0.03 | LDA only |
| 58 | *Caesio suevica* | Caesionidae | 0.04 | 0.01 | 0.03 | GLMM only |
| 59 | *Siganus stellatus* | Siganidae | 0.03 | 0.03 | 0.03 | Low consensus |
| 60 | *Lethrinus mahsena* | Lethrinidae | 0.01 | 0.05 | 0.03 | LDA only |
| 61 | *Aluterus scriptus* | Monacanthidae | 0.03 | 0.03 | 0.03 | Low consensus |
| 62 | *Ctenochaetus striatus* | Acanthuridae | 0.02 | 0.03 | 0.03 | Low consensus |
| 63 | *Odonus niger* | Balistidae | 0.02 | 0.04 | 0.03 | LDA only |
| 64 | *Calotomus viridescens* | Scaridae | 0.03 | 0.02 | 0.02 | Low consensus |
| 65 | *Scarus niger* | Scaridae | 0.02 | 0.03 | 0.02 | Low consensus |
| 66 | *Pseudochromis springeri* | Pseudochromidae | 0.02 | 0.02 | 0.02 | Low consensus |
| 67 | *Chlorurus sordidus* | Scaridae | 0.02 | 0.03 | 0.02 | Low consensus |
| 68 | *Caesio lunaris* | Caesionidae | 0.03 | 0.02 | 0.02 | Low consensus |
| 69 | *Pomacentrus trichrourus* | Pomacentridae | 0.01 | 0.04 | 0.02 | LDA only |
| 70 | *Cetoscarus bicolor* | Scaridae | 0.02 | 0.03 | 0.02 | Low consensus |
| 71 | *Hipposcarus harid* | Scaridae | 0.02 | 0.03 | 0.02 | Low consensus |
| 72 | *Zebrasoma xanthurum* | Acanthuridae | 0.02 | 0.02 | 0.02 | Low consensus |
| 73 | *Thamnaconus erythraeensis* | Monacanthidae | 0.01 | 0.03 | 0.02 | LDA only |
| 74 | *Argyrops megalommatus* | Sparidae | 0.01 | 0.03 | 0.02 | Low consensus |
| 75 | *Pygoplites diacanthus* | Pomacanthidae | 0.02 | 0.02 | 0.02 | Low consensus |
| 76 | *Pomacanthus maculosus* | Pomacanthidae | 0.02 | 0.01 | 0.02 | Low consensus |
| 77 | *Sufflamen albicaudatum* | Balistidae | 0.02 | 0.02 | 0.02 | Low consensus |
| 78 | *Chlorurus genazonatus* | Scaridae | 0.01 | 0.03 | 0.02 | Low consensus |
| 79 | *Pseudochromis fridmani* | Pseudochromidae | 0.01 | 0.03 | 0.02 | Low consensus |
| 80 | *Synodus variegatus* | Synodontidae | 0.02 | 0.01 | 0.02 | Low consensus |
| 81 | *Cheilinus lunulatus* | Labridae | 0.01 | 0.02 | 0.02 | Low consensus |
| 82 | *Balistapus undulatus* | Balistidae | 0.02 | 0.02 | 0.02 | Low consensus |
| 83 | *Sargocentron caudimaculatum* | Holocentridae | 0.01 | 0.02 | 0.02 | Low consensus |
| 84 | *Acanthurus nigrofuscus* | Acanthuridae | 0.02 | 0.01 | 0.02 | Low consensus |
| 85 | *Dascyllus marginatus* | Pomacentridae | 0.01 | 0.02 | 0.02 | Low consensus |
| 86 | *Cheilinus abudjubbe* | Labridae | 0.01 | 0.02 | 0.01 | Low consensus |
| 87 | *Coris caudimacula* | Labridae | 0.01 | 0.02 | 0.01 | Low consensus |
| 88 | *Gymnothorax undulatus* | Muraenidae | 0.01 | 0.02 | 0.01 | Low consensus |
| 89 | *Oxycheilinus digramma* | Labridae | 0.01 | 0.02 | 0.01 | Low consensus |
| 90 | *Platax orbicularis* | Ephippidae | 0.01 | 0.02 | 0.01 | Low consensus |
| 91 | *Pervagor randalli* | Monacanthidae | 0.02 | 0.01 | 0.01 | Low consensus |
| 92 | *Bodianus opercularis* | Labridae | 0.00 | 0.03 | 0.01 | Low consensus |
| 93 | *Centropyge multispinis* | Pomacanthidae | 0.02 | 0.01 | 0.01 | Low consensus |
| 94 | *Rhinecanthus assasi* | Balistidae | 0.01 | 0.01 | 0.01 | Low consensus |
| 95 | *Cephalopholis oligosticta* | Serranidae | 0.02 | 0.00 | 0.01 | Low consensus |
| 96 | *Larabicus quadrilineatus* | Labridae | 0.01 | 0.02 | 0.01 | Low consensus |
| 97 | *Cirrhilabrus blatteus* | Labridae | 0.00 | 0.02 | 0.01 | Low consensus |
| 98 | *Thalassoma rueppellii* | Labridae | 0.01 | 0.01 | 0.01 | Low consensus |
| 99 | *Chromis pelloura* | Pomacentridae | 0.00 | 0.02 | 0.01 | Low consensus |
| 100 | *Aetobatus ocellatus* | Myliobatidae | 0.01 | 0.01 | 0.01 | Low consensus |
| 101 | *Chromis dimidiata* | Pomacentridae | 0.00 | 0.02 | 0.01 | Low consensus |
| 102 | *Pseudochromis pesi* | Pseudochromidae | 0.01 | 0.01 | 0.01 | Low consensus |
| 103 | *Anampses twistii* | Labridae | 0.01 | 0.01 | 0.01 | Low consensus |
| 104 | *Gymnothorax elegans* | Muraenidae | 0.00 | 0.01 | 0.01 | Low consensus |
| 105 | *Pseudocheilinus evanidus* | Labridae | 0.01 | 0.01 | 0.01 | Low consensus |
| 106 | *Parapercis somaliensis* | Pinguipedidae | 0.00 | 0.01 | 0.01 | Low consensus |
| 107 | *Paracaesio sordida* | Lutjanidae | 0.00 | 0.01 | 0.01 | Low consensus |
| 108 | *Cephalopholis sexmaculata* | Serranidae | 0.00 | 0.01 | 0.00 | Low consensus |
